# A new bright future for organic light-emitting transistors

**DOI:** 10.1016/j.xinn.2025.100994

**Published:** 2025-06-13

**Authors:** Karl Leo

**Affiliations:** 1 Dresden Integrated Center for Applied Physics and Photonic Materials (IAPP) and Institute for Applied Physics, Technische Universitaet Dresden, 01062 Dresden, Germany

## Main text

The rapid advancement of organic semiconductors has fueled the remarkable growth of organic electronic devices, including organic light-emitting diodes (OLEDs), organic field-effect transistors (OFETs), and organic photovoltaics (OPV). Looking ahead, the evolution of organic optoelectronic devices is moving toward cost-effective, highly integrated, and flexible solutions with multifunctional capabilities, further expanding the range of potential applications.

**Figure 1:**
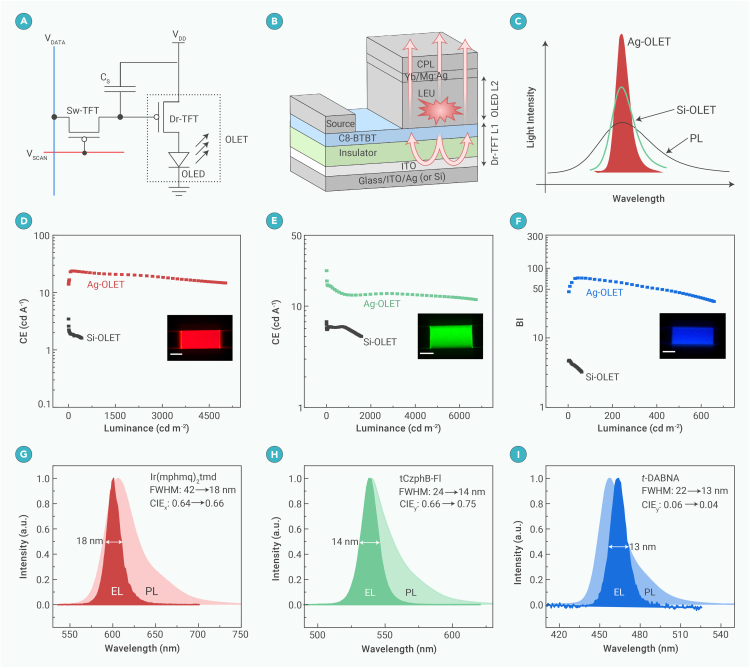
OLET device structure with intrinsic multiple-order microcavities and the optoelectronic performance of red, green, and blue OLETs (A) Schematic pixel circuit of traditional AM-OLED and OLET. (B) Simplified OLET structure, consisting of top-emitting OLED based on Ag or Si substrate-based TFT with intrinsic microcavity. (C) Illustration of spectral narrowing and intensity enhancement in Ag-OLET and Si-OLET. (D–F) CE/BI-luminance curves of RGB-OLETs; the insets show the emission photographs of the OLETs. (G–I) Comparison of the PL spectra of RGB emissive materials Ir(mphmq)_2_tmd, tCzphB-Fl, and *t*-DABNA with intrinsically narrow emission in dilute solutions and the spectra of RGB-OLETs with microcavity effects.

Organic light-emitting transistors (OLETs) represent a novel class of devices combining the gate modulation functionality of OFETs with the light-emitting properties of OLEDs, demonstrating significant promise for efficient next-generation displays, smart sensing, and optical communication systems (Figure 1A). However, OLET development has lagged behind other organic electronic devices due to the lack of suitable active materials that can simultaneously achieve high charge carrier mobility and strong solid-state emission, as well as the absence of mature device fabrication technologies. In recent years, substantial progress has been made in developing high-mobility emissive organic semiconductors and enhancing OLET device performance.^1^ Notably, the gate modulation capability of OLETs has been shown to significantly improve electric-to-optical conversion efficiency, surpassing that of conventional OLEDs. An external quantum efficiency (EQE) exceeding 20% has been proposed, originating from a novel vertical OLET structure.^2^ Moreover, OLETs have exhibited additional functionalities, such as optically switchable properties^3^ and intrinsic linear polarization,^4^ further expanding their potential applications.

Narrow electroluminescent emission is highly desirable in various fields, including high-resolution displays, optical communication, and phototherapy. It is challenging to achieve for OLEDs due to the usually broad emission spectra of organic emitters. A full width at half maximum (FWHM) of OLEDs ranging from 20 to 70 nm has been achieved by extensive optimization of the emitter molecular structures or fine-tuning of device architecture with resonance cavities (which is challenging to achieve in an OLET structure).

Recently, exciting research results on narrow-emission, high-efficiency OLETs published in *Nature Materials* demonstrate that the width of the electroluminescent spectrum could be efficiently reduced by an innovative OLET architecture that integrates a stacked planar OFET and OLED. This effect is attributed to the intrinsic multiple-order microcavities within the structure (Figure 1B).^5^ In their design, a fully reflective Ag mirror or a semi-reflective Si substrate is employed, while a semi-transparent Yb/Mg:Ag top electrode serves as the out-coupling interface, forming the two parallel ends of a Fabry-Pérot cavity. This configuration facilitates multiple photon oscillations within the optical microcavity, creating narrow and efficient emission from the top electrode. As a result, both the emission efficiency and spectral purity are significantly improved (Figure 1C). By strategically selecting energy-level-matched device structures and luminescent materials, the researchers achieved outstanding current efficiencies (or blue index [BI] values for blue) of 26.3, 37.3 cd A^−1^, and 72.6 for red, green, and blue devices, respectively (Figures 1D–1F). Additionally, the OLET devices exhibit excellent electrical performance, with a low turn-on voltage of 3.5 V and a high switching ratio of 10^5^, demonstrating strong gate modulation capabilities and low energy consumption. To validate the universality of this spectral narrowing strategy, the team introduced various luminescent molecules with different emission mechanisms into the system. Notably, the spectral widths of narrowband RGB emitters *t*-DABNA, tCzphB-Fl, and Ir(mphmq)_2_tmd are reduced from 22, 24, and 42 nm in photoluminescence (PL) to an impressive 13, 14, and 18 nm, respectively. Similarly, the FWHMs of the thermally activated delayed fluorescence (TADF) material DMAC-DPS are narrowed from 64 to 23 nm. The phosphorescent emitters Ir(ppy)_2_acac and Ir(piq)_2_acac are improved from 50 and 55 to 16 and 19 nm, respectively (Figures 1G–1I). The optimizations resulted in an exceptionally wide color gamut of 97% in the BT.2020 color space. These results represent a significant advancement in the OLET field, demonstrating the superior performance enabled by the intrinsic microcavity effect, paving an effective way for the development of high-efficiency, narrow-emission organic optoelectronic devices.

In summary, this work introduces an innovative strategy for achieving narrow electroluminescent emission while simultaneously enhancing electric-to-optical conversion efficiency by leveraging the intrinsic microcavity effect in OLETs. The microcavity structure not only enables precise optical tuning but also optimizes exciton dynamics, resulting in improved light extraction efficiency and spectral purity. These findings represent a significant milestone in the advancement of OLET technology, establishing it as a transformative platform for organic optoelectronic devices.

The demonstrated approach is expected to accelerate the practical implementation of OLETs in next-generation display technologies, where high color purity and energy efficiency are crucial. Additionally, the ability to finely tune emission characteristics through device architecture design makes OLETs highly promising for optical communication systems, enabling high-speed, low-loss signal transmission and paving the way for a bright future in the development of OLET technology.

## Funding and acknowledgments

I thank Prof. Wenping Hu from Tianjing University and Prof. Huanli Dong from the Institute of Chemistry, Chinese Academy Sciences, and their co-workers for the very helpful discussions.

## Declaration of interests

K.L. is a shareholder of Credoxys GmbH, an OLED dopant manufacturer, and beeOLED, an OLED emitter manufacturer.
